# Supplementary figures and images for: Alternative to ZnO to establish balanced intestinal microbiota for weaning piglets
Source: PLoS One. 2022 Mar 17;17(3):e0265573. doi: 10.1371/journal.pone.0265573 (PMC8929640; doi:10.1371/journal.pone.0265573)

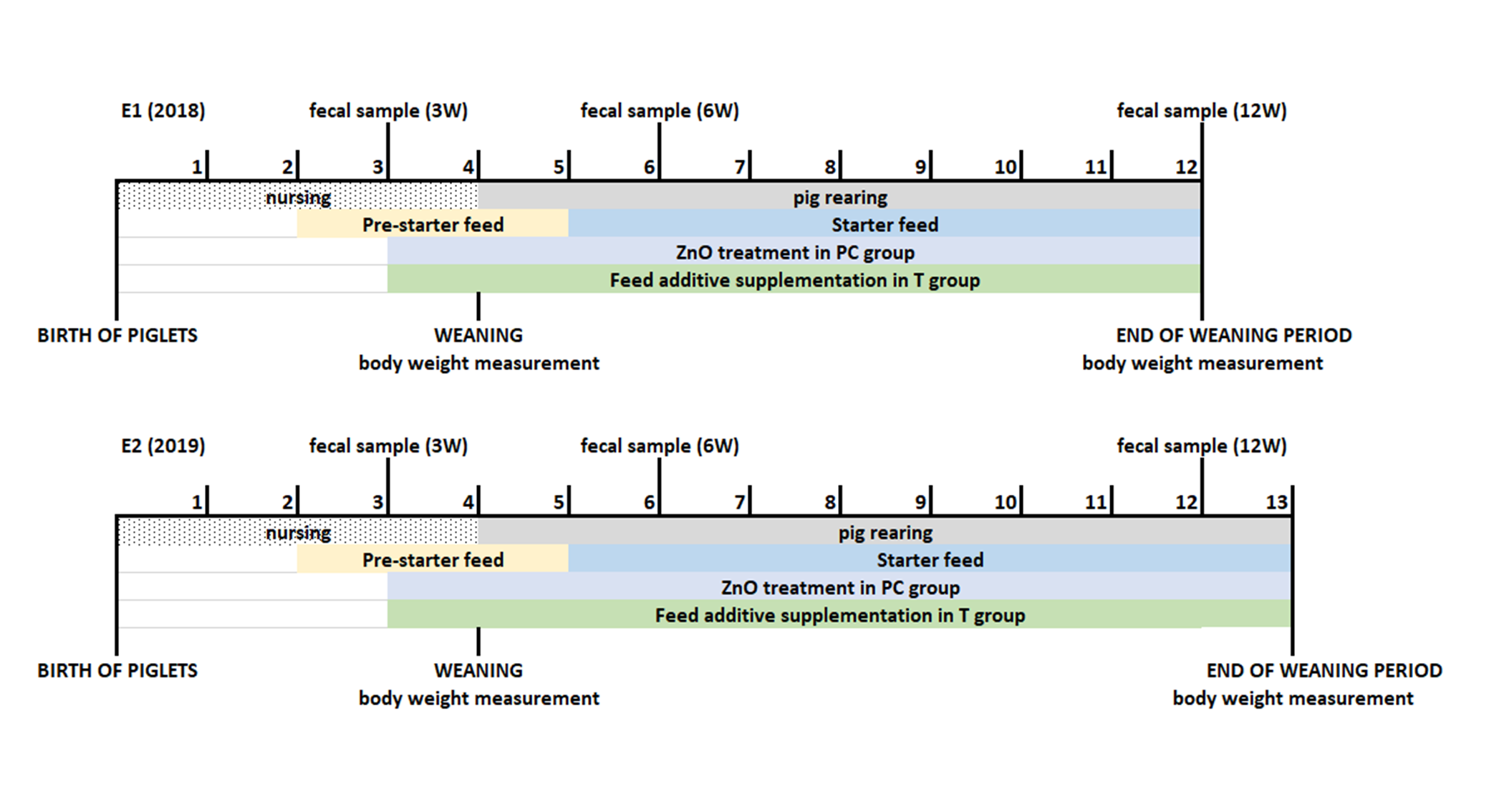

Supplement: S1 Fig — The numbers (1–13) represent the average age of the animals in weeks. The dotted part represents the nursing period. The gray shaded part indicates the continuous monitoring of feed consumption. The colored areas indicate the type of feeds and treatments in the T and PC groups. (TIF) [file pone.0265573.s001.tif]

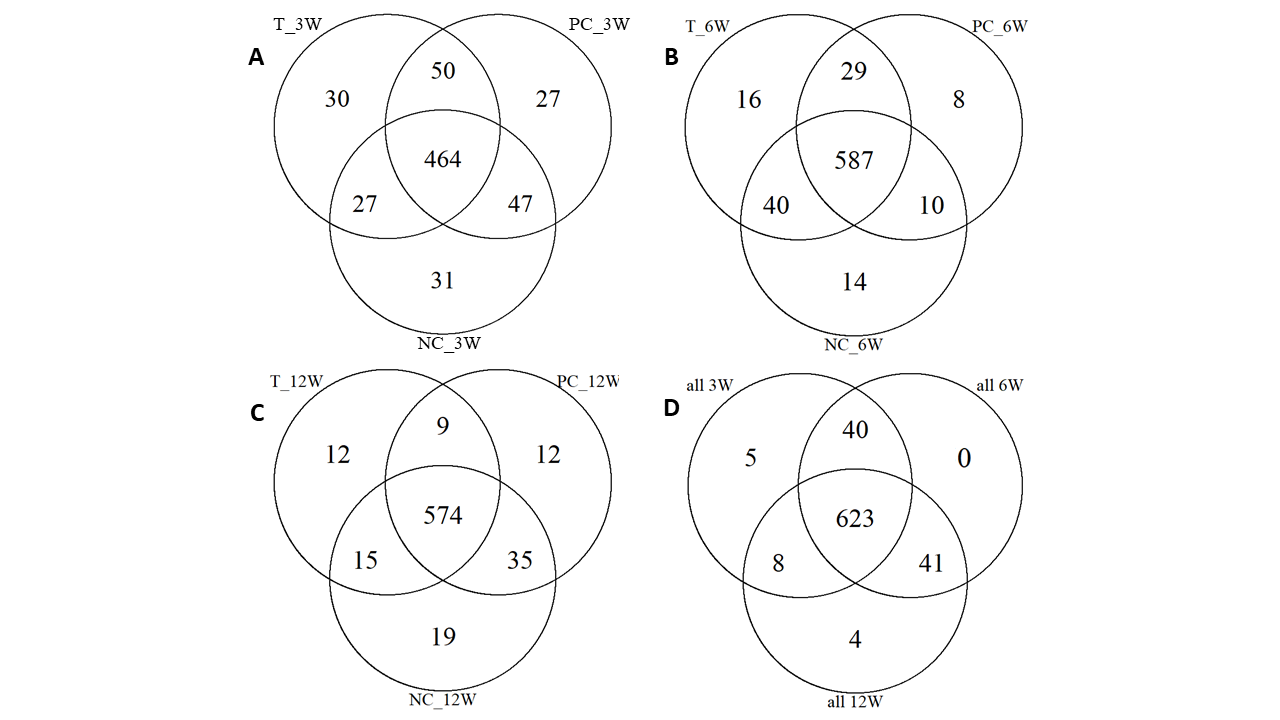

Supplement: S2 Fig — A, B and C: differences between treatments at 3, 6, and 12 weeks. D: age-related differences. (TIF) [file pone.0265573.s002.tif]

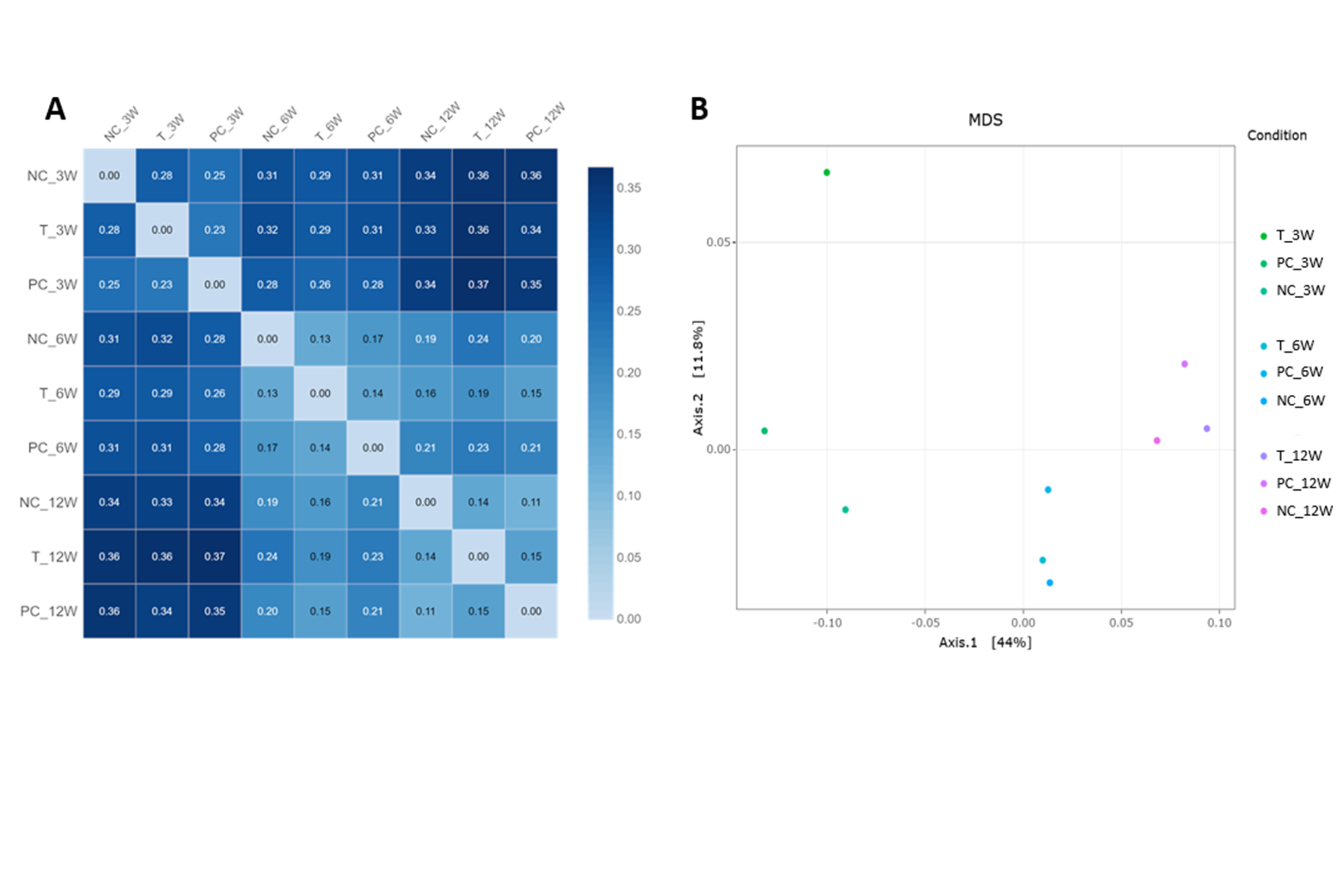

Supplement: S3 Fig — (A) Heatmap highlighting the Jaccard diversity index among the microbial populations. The higher the color intensity, the lower the similarity between the pairs. (B) Multi-dimensional scaling of the data set. The dots of green, blue, and purple represent 3, 6, and 12 weeks, respectively. (TIF) [file pone.0265573.s003.tif]

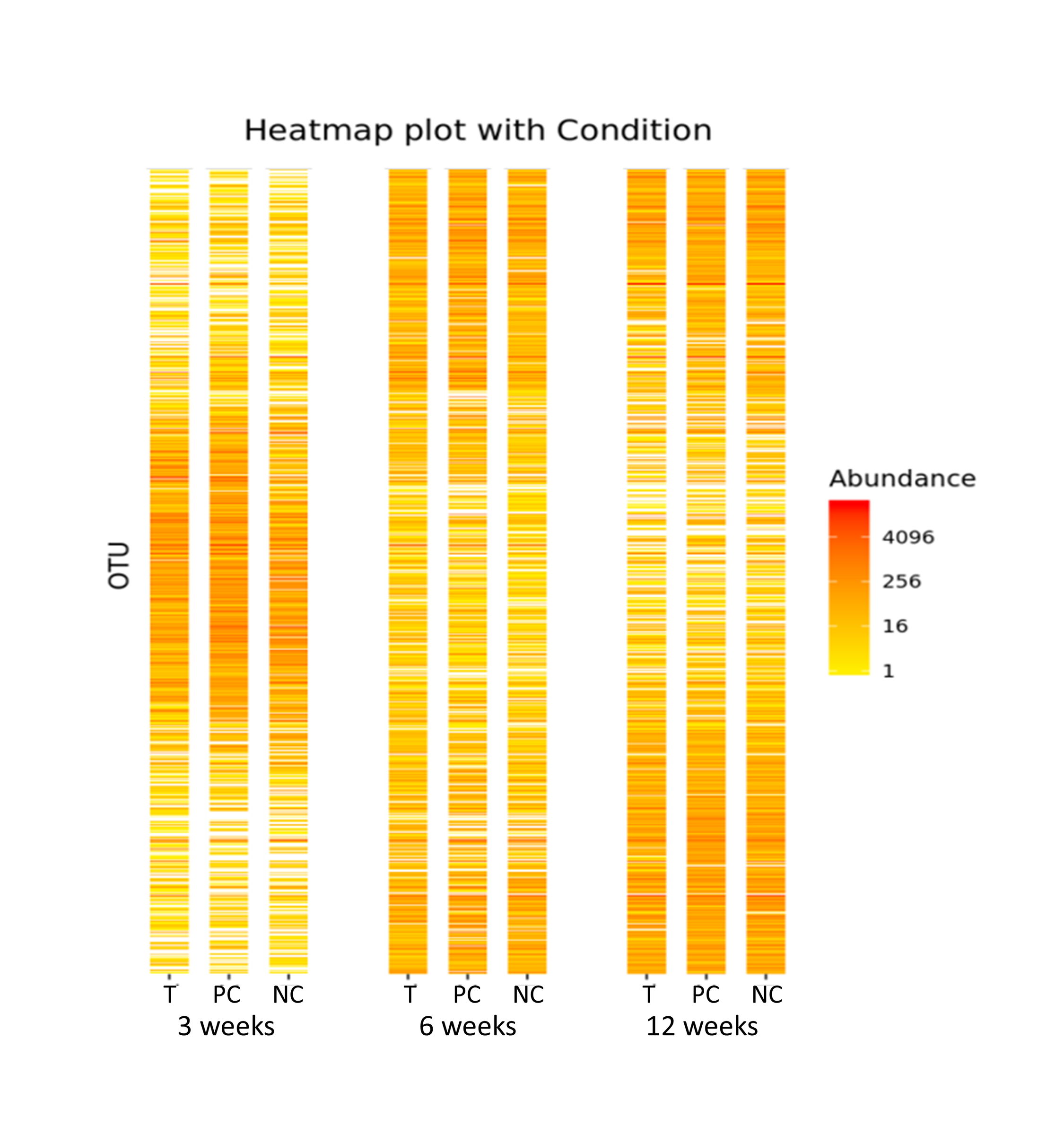

Supplement: S4 Fig — (TIF) [file pone.0265573.s004.tif]
