# Supplementary material for: Alternative to ZnO to establish balanced intestinal microbiota for weaning piglets
Source: PLoS One. 2022 Mar 17;17(3):e0265573. doi: 10.1371/journal.pone.0265573 (PMC8929640; doi:10.1371/journal.pone.0265573)
Supplement: Attachment — Submitted filename: PONE-D-2136256 Ghonimy letter.doc [file pone.0265573.s010.doc]

Use the term “diarrhea” only for your recommendation for the future studies, since your experimental design does not contain diarrhea treatment.

L65, add the hypothesis

L 66-68, I suggest you using this rephrased paragraph

The objective of this study was to evaluate the effect of phyto- and pre-biotic feed additives (curcumin, wheat germ and chicory) as alternatives to ZnO on intestinal microbiota composition and growth performance in weaned piglets.

L 71, provide a figure describing your experimental design.

L 229, delete “**with different”**

L 58-61, it is already stated at introduction section.

L 363, write it in more simple way

L 354-369, it is too long introduction till you started to explain your results, I suggest you summarizing it.

L 377, explain why your results are in agreement with the literature.

For discussion part, try to give explanation why your results are similar to the literature results, such as mechanism or physiological effects on bacterial functions. For example, phenol compound affects cell wall of gram-negative bacteria. Or the effect of poly saccharides or your study bioactive compounds etc….

L 481, summarize the conclusion stating the most important results based on your measurements, in addition to the future application or invention which may be used based on your results.
